# New Medical Journal

**Published:** 1852-10

**Authors:** 


*New Medical Journal.*—*The Philadelphia Medical and Surgical Journal*, is the title of a new monthly, with the name of James Bryan, M. D., as editor. Dr. B. has contributed largely to the medical periodical literature of this country for several years past. Some valuable communications from his pen have appeared in this Journal. He has our best wishes in behalf of his new enterprise.

